# Application of Nanotechnology for Sensitive Detection of Low-Abundance Single-Nucleotide Variations in Genomic DNA: A Review

**DOI:** 10.3390/nano11061384

**Published:** 2021-05-24

**Authors:** Mahwash Mukhtar, Saman Sargazi, Mahmood Barani, Henning Madry, Abbas Rahdar, Magali Cucchiarini

**Affiliations:** 1 Faculty of Pharmacy, Institute of Pharmaceutical Technology and Regulatory Affairs, University of Szeged, 6720 Szeged, Hungary; mahwash.mukhtar@szte.hu; 2 Cellular and Molecular Research Center, Resistant Tuberculosis Institute, Zahedan University of Medical Sciences, Zahedan 98167-43463, Iran; sgz.biomed@gmail.com; 3 Department of Chemistry, Shahid Bahonar University of Kerman, Kerman 76169-14111, Iran; mahmoodbarani7@gmail.com; 4 Center of Experimental Orthopaedics, Saarland University Medical Center, D-66421 Homburg/Saar, Germany; henning.madry@uks.eu; 5 Department of Physics, Faculty of Science, University of Zabol, Zabol 538-98615, Iran

**Keywords:** SNP, nanotechnology, genomic DNA, detection

## Abstract

Single-nucleotide polymorphisms (SNPs) are the simplest and most common type of DNA variations in the human genome. This class of attractive genetic markers, along with point mutations, have been associated with the risk of developing a wide range of diseases, including cancer, cardiovascular diseases, autoimmune diseases, and neurodegenerative diseases. Several existing methods to detect SNPs and mutations in body fluids have faced limitations. Therefore, there is a need to focus on developing noninvasive future polymerase chain reaction (PCR)–free tools to detect low-abundant SNPs in such specimens. The detection of small concentrations of SNPs in the presence of a large background of wild-type genes is the biggest hurdle. Hence, the screening and detection of SNPs need efficient and straightforward strategies. Suitable amplification methods are being explored to avoid high-throughput settings and laborious efforts. Therefore, currently, DNA sensing methods are being explored for the ultrasensitive detection of SNPs based on the concept of nanotechnology. Owing to their small size and improved surface area, nanomaterials hold the extensive capacity to be used as biosensors in the genotyping and highly sensitive recognition of single-base mismatch in the presence of incomparable wild-type DNA fragments. Different nanomaterials have been combined with imaging and sensing techniques and amplification methods to facilitate the less time-consuming and easy detection of SNPs in different diseases. This review aims to highlight some of the most recent findings on the aspects of nanotechnology-based SNP sensing methods used for the specific and ultrasensitive detection of low-concentration SNPs and rare mutations.

## 1. Introduction

Modern diagnostic methods have shown better performance than conventional methods. Recently, the focus has moved beyond the conventional clinical therapies and gravitated toward individualized therapies, termed “personalized medicine” [1,2,3,4,5]. Personalized medicine is a wide-ranging and expeditiously advancing field in health care that provides remarkable access to each patient’s unique genetic, genomic, and clinical characteristics [6]. This new trend in medicine also, called “precision medicine”, addresses distinctive molecular defects present in thousands of genetic abnormalities via the application of various technologies [7].

Many efforts have been made in the last 20 years to chart more than 3 million single-nucleotide polymorphisms (SNPs) in the human genome [8]. These genetic variations were assumed to be not deleterious to organisms and were primarily studied because of their clinical significance and application in personalized medicine [9]. In this regard, broad clinical applications of personalized medicine have been reported for SNPs, such as risk assessment, screening of different multifactorial diseases, diagnosis, prognosis, and patient stratification [9,10]. This implies that SNPs could serve as valuable biomarkers for predicting patient’s response to treatment, adverse effects of drugs, and drug resistance [9]. On the other hand, point mutations are rather subtle changes in DNA; hence, their detection is challenging [11]. This problem has gained much attention, and due to the importance of identifying SNPs in humans, multiple genotyping methods have been developed. However, similar to the SNPs, precise detection of low-abundance somatic point mutations is a critical step in characterizing disease genomics [12].

Conventional genotyping methods or more advanced array comparative genomic hybridization methods have proven to be reliable genomic technologies used to evaluate disease incidence and to reveal the impact of SNPs on disease progression [13,14]. Some serological tests such as enzyme-linked immunosorbent assay (ELISA), polymerase chain reaction (PCR), real-time reverse transcription PCR (RT-PCR), luciferase immunoprecipitation system (LIPS), next-generation sequencing (NGS), and loop-mediated isothermal amplification (LAMP) have already been used for the diagnosis of diseases, identification of SNPs, and/or rapid detection of pathogens [15,16,17]. However, they are less sensitive, time-consuming, and relatively expensive for such purposes [18]. Among these, the tetra amplification refractory mutation system-PCR (T-ARMS-PCR), PCR-restriction fragment length polymorphism (PCR-RFLP), RT-PCR high-resolution melt (HRM), and PCR-single-strand conformation polymorphism (PCR-SSCP) are independent diagnostic methods widely used in SNP genotyping. However, the efficacy of these techniques is hindered by many optimizations and lack of experience [19,20].

Next-generation sequencing (NGS) is a parallel sequencing method developed to extend the resolution and scale of genomic investigations. Despite recent advancements made using NGS platforms, a major obstacle in the application of this technique for SNP detection remains in data processing steps [21]. It has been reported that most mainstream NGS technologies exploit short-read lengths, which limits the detection of structural variations/mutations and the ability to conduct de novo sequencing [22]. Wang et al., showed that NGS has lower sequence coverage and poor SNP-detection capability in the gene’s regulatory regions [23]. Furthermore, several NGS systems make use of microtiter plates for sample partitioning. These methods are instrumentally complex and limited in their partitioning capacity [24]. Hence, the use of nanoscale droplets has been suggested to yield large-scale partitioning more efficiently and rapidly [25].

As a burgeoning field, nanotechnology uses nanoscale materials for many biomedical applications, including gene therapy drug delivery, tissue engineering, etc. [26,27,28,29,30]. Controlling the size of nanomaterials contributes to the emergence of new microscopic properties [31,32,33]. For example, a nanofluid is a fluid containing nanometer-sized particles, called nanoparticles. These fluids are engineered colloidal suspensions of nanoparticles in a base fluid. The nanoparticles used in nanofluids are typically made of metals, oxides, carbides, or carbon nanotubes [34,35,36,37,38,39]. This is interesting because DNA has physical properties that can be utilized for the bottom-up construction of nanostructures [40]. The high flexibility of DNA as a single strand enables this molecule to increase its rigidity by 50 fold as a double strand, creating the basis for using this genetic material as a nanoscale building block [40,41].

Lately, nanotechnology has revolutionized our ability to better detect SNP mutations and understand the genetic basis of numerous complex and common disorders. Thanks to their easy operation and high specificity, the application of these nanobased devices has been spotlighted as ideal tools for detecting nucleic acids, SNPs, or point mutations. In this context, Li and colleagues [42] designed branched DNA structures with colored fluorophores, named nanobarcodes, and used them to detect the presence or absence of pathogenic DNA fragments. Guo et al. [43] developed a label-free colorimetric system to detect SNPs using novel hemin–graphene hybrid nanosheets with intrinsic peroxidase-like activity. Goldsworthy and coworkers designed smart nanodevices based on RNA binding aptamers as a novel system for nucleic acid detection [44]. Xu’s research team [45] developed shell-engineered nanoparticles (NPs) coated with silver (Ag) and gold (Au) and observed a significant improvement in the sensitivity of PCR-based DNA detection. Patolsky et al. [46] incorporated biotin labels into DNA replicas associated with magnetic particles as a nanodevice for accurate DNA detection. The combination of NGS and nanotechnology for detecting SNPs have also gained much attention. In this respect, Siravegna and colleagues combined BEAMing (nanobeads, emulsion, amplification, and magnetics), droplet digital PCR, NGS, and bioinformatics analyses to accurately genotype genetic variations associated with colorectal cancer during treatment with epithelial growth factor receptor (EGFR)–specific antibodies [47]. Magnetic nanoparticles (MNPs) have been shown to increase the throughput for NGS [48]. Baker et al. prepared NGS libraries for constructing the cystic fibrosis transmembrane conductance regulator (*CFTR*) gene variations using Illumina MiSeq NGS system and magnetic beads [49]. Hertz and coworkers efficiently used MNPs and the Illumina MiSeq NGS technique for genotyping DNA variations that contributed to the occurrence of sudden infant death syndrome [50]. Such methodologies have also shown promising implications for designing personalized treatments of diverse diseases.

Furthermore, nanotechnology-enhanced electrochemical sensors have shown great promise for detecting mismatched base pairs in DNA [51,52]. Compared with previous works, Hwang et al. [53] showed that using a DNA tweezers probe with high-quality graphene field-effect transistor enhances the sensitivity of SNP detection by more than 1000 fold. Designing DNA carriers containing only one single base difference can help to recognize DNA strands. Therefore, it is suggested as an alternative strategy toward detecting SNPs or mutations at a single-molecule level [54].

Single genetic variations play a crucial role as molecular biomarkers in medical and diagnostic applications.   In the present article, we attempted to provide a comprehensive review of the role of nanotechnology in detecting SNPs for their application in personalized medicine. 

## 2. What Are SNPs?

Genetic variations in the human genome are considered an emerging resource for studying complex diseases. As the simplest form of DNA variation among individuals, SNPs have gained much attention for better understanding the etiology of complex diseases [8,55]. SNPs might influence divergent traits, which is commonly the basis of predicting traits or susceptibility to diseases. They can be transitions or transversions at the frequency of approximately one in a thousand base pairs (bp) [56]. It has been reported that almost half of SNPs occur in the noncoding regions of DNA, 25% are synonymous or silent mutations, and the remaining 25% are missense mutations [57]. It was previously hypothesized that silent SNPs do not influence gene function and haplotype because they do not change the encoded amino acids [8], but this hypothesis was later proved wrong [58]. Nonsynonymous SNPs, however, affect gene expression, stability of messenger RNA (mRNA), subcellular localization of mRNA/proteins, and the translation process as well [8,59,60,61]. By definition, an SNP has a minor allele frequency >1% in a population [62]. In other words, SNPs arise due to the presence of point mutations in populations.

## 3. Clinical Significance of SNP Mutations

SNPs, in the presence or absence of other risk factors, are responsible for the susceptibility of individuals to multifactorial diseases and may determine the phenotypic expression of such diseases [63,64]. This has made predictive SNPs excellent tools for medical testing and a safer individualized prognostic marker [65]. Nevertheless, genetic variations discovered by genome-wide association studies (GWAS) constitute a minor fraction of SNPs of complex traits in humans, and the remaining heritability can be explained by incomplete linkage disequilibrium between genotyped SNPs and casual variations having low allele frequencies [66].

Essentially, the presence of a heterozygote allele may not change the risk of developing a disease, but a homozygote allele of the same SNP significantly affects susceptibility to a complex polygenic disease [67]. Recently, germline and somatic sequence SNPs in coding and/or noncoding DNA regions have been largely investigated for their role in the onset of cancer [68,69], inflammatory diseases [70,71], eye diseases, cardiovascular diseases [72,73], kidney diseases, endocrine disorders, skin diseases [74,75], psychological diseases and anxiety disorders, gynecological diseases, etc. Interestingly, a large number of population-based studies reported an association between the studied variant and disease risk. Synergistic effects of the risk alleles of some SNPs with environmental factors on the susceptibility to such diseases were also reported in several studies [76,77]. Point mutations have been introduced as a source of de novo genetic diseases [78]. Therefore, gaining a better understanding of the role of genetic variations in the etiology of the conditions above will expand our horizons for designing such curative strategies.

## 4. Detection of SNPs

### 4.1. Current Methods

SNPs are often hard to differentiate from other wild-type DNAs because only one base pair is changed in the DNA sequence [79]. The detection in such cases needs substantial amplification by sensitive techniques. However, factors such as the utilization of DNA polymerases, several primers, time-consuming strict working environment, and the possibility of contamination make techniques such as PCR a hectic procedure [80].

Unlike traditional DNA restriction fragment length polymorphisms (RFLPs) or polymerase chain reaction (PCR)–based markers that depend on unified detection methods, SNPs can be identified using various methods, with new ones emerging every year. Around 20 different SNP detection approaches have been identified so far, consisting of various combinations of different allele-discrimination chemistries and signal detection systems [81]. SNP detection strategies were initially focused on gel electrophoresis, such as cleaved amplified polymorphic sequence (CAPS) labeling [82], allele-specific PCR (AS-PCR) [83], PCR-single-strand conformation polymorphism (PCR-SSCP) [84], and denatured gradient gel electrophoresis (DGGE) [85], among others. These techniques, however, were not appropriate for large-scale procedures, and only a small percentage of SNPs could be identified, with low-frequency signals being ignored. SNPs were identified using greater and automatized methods in addition to gel-electrophoresis-based methods, such as DNA sequencing and DNA microarray [86], denaturing high-performance liquid chromatography (DHPLC) [87], matrix-assisted laser desorption ionization-time of flight (MALDI-TOF) [88], and high-resolution melting (HRM) [89]. These low-throughput approaches, on the other hand, have a high instrument and sample preparation demands. Of note, DNA sequencing and DNA microarray are the most commonly used methods for SNP identification these days because they are high-throughput and cost-effective approaches. Nevertheless, there is a need for highly specific detection of SNPs, and nanotechnology-based methods can be a final solution for this purpose.

### 4.2. Nanotechnology-Based Methods

**Figure 1:**
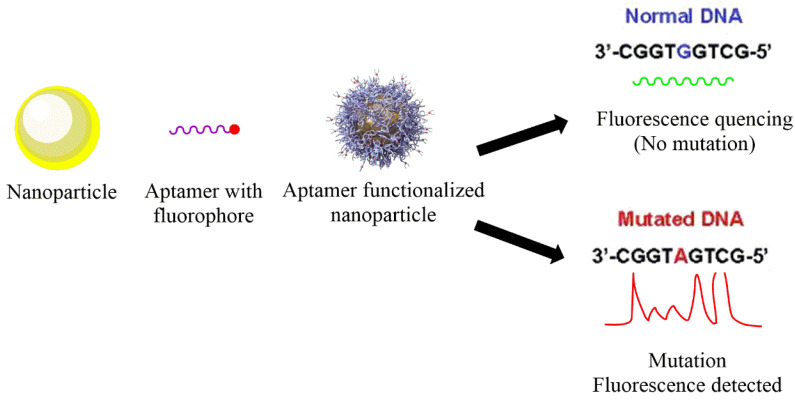
General representation of method used for the detection of single-nucleotide polymorphisms based on nanotechnology.

Nanotechnology holds promising results in identifying SNPs by various principles, such as partial aggregation upon recognizing target DNA. Moreover, the signal amplification can be solely achieved using optical nanocarriers that may further be improved using hybrid techniques, such as merging the sensing methods with nanotechnology. Au, silica, Ag, graphene, and quantum-based nanostructures have been widely studied in diagnostic applications in the past. Hence, owing to their positive outcomes, they are also being explored in recognition of SNPs. A general diagram representing the basic idea of SNP detection is shown in Figure 1.

#### 4.2.1. Ultrasensitive Hybrid Nanotechnology

Lately, various SNPs detection procedures have been introduced. Few of these are surface-enhanced Raman scattering, high-resolution DNA melting, hybridization chain reactions, and single molecular fluorescence [90,91,92,93]. But all the techniques lack precision and sensitivity to the specific enzyme recognition site. Currently, procedures such as ligase-based approaches are being studied as they may work with various targets with significant SNPs detection. Ultrasensitive detection of SNPs makes it possible not only to diagnose the disease but also to predict the relapse [94]. Ultra-sensitivity can be achieved by coupling the NPs to the ligand-based procedures. MNPs are popular in this regard because of their stability and homogenous dispersions. Moreover, they are also capable of amplification of enzymatic signaling [95].

One such research focused on the MNPs for the detection of SNP. MNP-signal-DNA-ligation was introduced to improve the target DNA to enzyme conversion ability. The MNP nanobead poly-enzyme enabled the signal amplification by producing copies of enzymes for each captured target DNA. The whole method improved the sensitivity and detection of cancer genetic mutations such as V-KI-RAS2 Kirsten rat sarcoma viral oncogene homolog (*KRAS*) gene mutations. These somatic mutations are common in lung and colorectal cancers and were used as a model DNA target in the study [96]. The method used in the research utilized the hybrid approach in which MNPs were combined with poly-enzyme nanobead signal amplification. The nanobeads were tagged with copies of horseradish peroxidase (HRP) to enhance the signal amplification power. The perfect-match target DNA presented biotinylated signal-DNA on the MNP to allow binding of neutravidin HRP. This method is an ultrasensitive approach to detect SNPs linked to cancer. Biotinylated signal-DNA and MNP-linked capture DNA sandwiched the target with high efficiency and performed ligation to improve the SNP detection. The results demonstrated that the procedure provided high discrimination between the perfect-match target DNA and its cancer-linked SNPs. It detected a 1 fM perfect-match target DNA in the presence of 100-fold single-base-mismatch targets.

Currently, isothermal amplification techniques are being exploited to target amplification for SNP recognition. One such technique is loop-mediated isothermal amplification (LAMP), which is highly specific and efficient in targeting amplification [97]. The method is also used in the characterization of a specific SNP by an allele-specific LAMP. This technique is being studied in drug resistance and the detection of complex disorders. Nanotechnology is being used to characterize specific DNA sequences with precision. Allele-specific LAMP amplification was used in a study, and Au nanoprobes were also used because of their optical properties. Au nanoprobes yield a different color in the absence and presence of complementary targets. Mismatched DNA aggregates Au nanoprobes creating blue color, whereas unaltered DNA does not, leading to red color. The research focused on the screening of the SNP related to lactose intolerance. The turnaround time for amplification was found to be 3 h in the presence of Au nanoprobes. The Au nanoprobes significantly interpreted the allele-specific amplified product in less than 15 min. This novel idea is an excellent approach for diagnosing the most commonly found intolerances in humans [98].

Similarly, nanotechnology was explored for the diagnosis of cancer. The blood of cancer patients has elevated levels of circulating DNA, which might be due to high cellular apoptosis and lysis of cancer cells resulting in a mixture of wild-type DNA along with mutant DNA [99]. The circulating tumor DNA (ctDNA) is the mutant DNA but can be present in various types of cancer. The extremely low levels of ctDNA are difficult to detect with accuracy amid the wild-type DNA concentrations. Hence, AuNPs were used along with MutS protein and a microcantilever to diagnose ctDNA. This specific enzyme is present in *Escherichia coli* and can bind with SNPs through a hydrogen bond by initiating the mismatch repair system. A simple adsorption procedure, molecular dynamic simulation, was used to bind the MutS protein to AuNPs. Moreover, a microcantilever resonator was used to detect the target by identifying the frequency changes and, hence, was employed as the bio-nanosensor. AuNPs amplified the mutant DNA signal on the microcantilever, which used thiolated DNAs as probe DNAs. The method efficiently detected the *KRAS* mutations from other ctDNAs. This specific oncogene is present in non-small-cell lung cancer. AuNPs-MutS significantly increased the mass of the cantilever in the presence of mutant DNA. The use of AuNPs improved the selectivity of the detection by using MutS. This novel technique accurately diagnosed the disease-related SNPs out of all the other wild-type DNAs [100].

Lately, an interesting technique called enzymatic biofuel cells (EBFCs) has gained attention in biosensing applications. EBFCs can generate electrical energy from biofuels [101]. EBFCs are easy to develop with simple instrumentation, but there is a limitation to their use because of low sensitivity toward targets. A recent study investigated the EBFCs integrated with a DNA amplification strategy [102]. The self-powered ultrasensitive biosensor for detecting SNPs was fabricated by combining DNA hybridization chain (HCR) and toehold-mediated strand displacement reaction (SDR). EBFCs were built of a glucose dehydrogenase bioanode and the capture probe of AuNPs. The use of AuNPs improved the amplification of the signal and facilitated higher sensitivity. DNA HCR and SDR reacted to produce a long double-helix chain in the presence of a target sequence. The electrostatic interaction between the double-helix chain and (Ru(NH_3_)_6_)^3+^ produced voltage in the circuit, exhibiting the detection of SNPs. The strategy efficiently identified the p53 gene fragment from wild sequences paving the way for the novel diagnostic platform.

Altogether, the technique might be an old concept, but exploiting nanotechnology has made it sensitive toward single-base mismatched DNA detection. This novel approach is gaining attention in diagnostic purposes and can be explored further by the attachment of functional moieties to improve sensitivity.

#### 4.2.2. Electrochemiluminescence Detection

**Figure 2:**
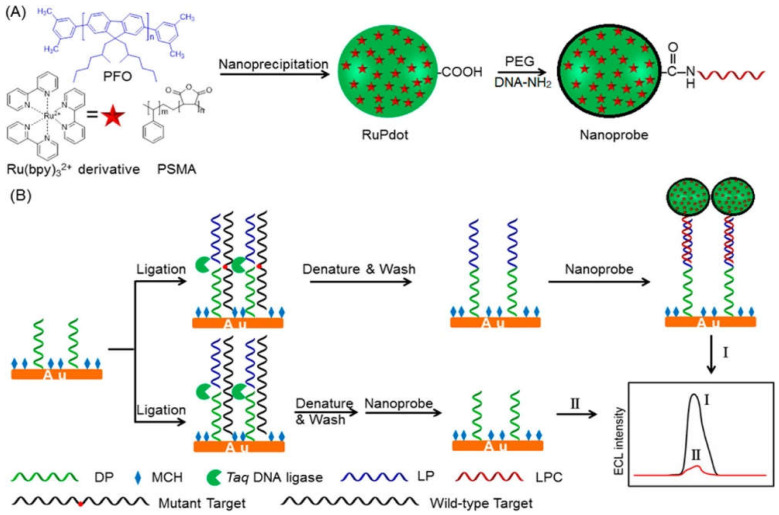
Schematic illustration of (**A**) the synthesis of a Ru(bpy)_3_^2+^-doped Pdots nanoprobe using PFO as a carrier and PSMA as a functional reagent, and (**B**) the detection of SNPs with nanoprobe and ligase detection reaction. Reprinted with permission from ref. [106]. Copyright 2017 American Chemical Society.

The classic electrochemistry approach used methods labeled with natural enzymes or electro-molecules to yield the amplification signal. Nevertheless, this conventional approach has problems of heavy background noise and nonspecificity [103]. Another main challenge with the method above is the identification of different types of single-base mismatches. Hence, the technique was modified to enhance sensitivity and diagnostic ability. A modified similar technique that enables the efficient detection of SNPs is electrochemiluminescence (ECL). The most commonly used luminophore is Ru(bpy)_3_^2+^ because of its high ECL efficiency [104]. However, to further improve the sensitivity of the ECL technique, ECL emission of Ru(bpy)_3_^2+^ needs to be enhanced [105]. Recently, NPs have gained interest in increasing the excitation rate and the emission factor of Ru(bpy)_3_^2+^ to help in better detection. The fluorescent nanomaterials are of special consideration in this regard due to their photostability and fast emission rate. Polymer dots (Pdots) have been widely studied in biosensing. They are advantageous over quantum dots (QDs) as they are nontoxic. A recent study combined the luminescent Pdots encapsulated with Ru(bpy)_3_^2+^ to develop the double enhanced ECL mechanism for detecting SNPs. Poly(9,9-dioctylfluorenyl-2,7-diyl) (PFO) was used as a carrier during the process of nanoprecipitation, poly(styrene-co-maleic anhydride) (PSMA) as the functional reagent, and Ru(bpy)_3_^2+^ as the ECL active molecule, as shown in Figure 2. Excited Pdots transferred resonance energy to Ru(bpy)_3_^2+^ that further improved the emission intensity and led to high sensitivity in biodetection. The DNA-functionalized Ru(bpy)_3_^2+^-doped Pdots were developed to detect the *KRAS* mutant gene. The Ru(bpy)_3_^2+^-doped Pdots had an average size of 20 nm. These novel ultrasensitive ECL nanoconjugates amplified the detection signal in the linear range from 1 fM to 1 nM [106].

Another novel strategy for detecting SNPs uses carbon nanostructures that can be in the form of carbon nanospheres, nanocapsules, nanotubes, or nanofibers. The graphene quantum dots (GQDs) were developed in a study to detect DNA damage. These GQDs were synthesized using any chemical reagent. Moreover, AuNPs were attached to the single-stranded DNA probe (cp53 ssDNA) to improve the ECL mechanism. These AuNP-ssDNAs, when attached to the GQDs, enhanced the ECL signal quenching of GQDs, and when these AuNP-ssDNAs were hybridized with target p53 DNA, they formed AuNP-dsDNAs leading to the recovery of the GQDs’ ECL. The developed nanosystem provided a sensor platform for the efficient detection of SNPs and the quantification of aptamer-specific biomolecules [107].

An interesting study emphasized the use of a label-free electrochemical biosensing method. The biosensor comprised the Ag/platinum (Ag/Pt) bimetallic nanoclusters fixed on the triplex DNA template. Ag/Pt NPs were used due to their high stability and synergistic effect compared with monometallic NPs. The X-shaped DNA probe was fabricated with the triplex Ag/Pt nanoclusters and locked nucleic acid (LNA). The triplex Ag/Pt nanoclusters were able to detect the SNPs related to β-thalassemia. The LNA modified X-shaped triplex Ag/Pt nanoclusters were attached to the surface of the Au electrode. This assembly dissociated in the presence of the target, causing the elevated signal that detected the variant allele frequency in β-thalassemia [108].

Lately, a single platform for genotyping the SNPs was reported in which modified NPs were utilized for electrochemical signals [109]. The linkers, cysteine and cysteamine hydrochloride, were used to modify AgNPs and AuNPs. The bimetallic NPs were used because of their high conductivity and enhanced electron transfer properties. The complementary monobases were anchored to the MNPs, and the mismatch targets were hybridized by the monobase-linked MNPs in the presence of DNA polymerase I. This hybridization was followed by the analytical signals for SNP genotyping, detected as electrooxidation signals of AgNPs and AuNPs. The polymerase enzyme stimulated the coupling of the mutant site of DNA (hemophilia gene sequence) to the monobase-linked MNPs. This method allowed the detection of complementary targets with a linear range of 20–1000 pM and 50–1500 pM of mutant DNA.

Similarly, chemiluminescence-based sensors have been explored encapsulated with luminol, which works as a chemiluminescence illuminant. Silicon material has been used in the past for antigen detection as a target-triggered signal chemiluminescence sensor. In the presence of a specific antigen, e.g., prostate specific antigen, the antigen and its aptamer are combined together. Once this happens, the aptamer mounted on the silica detaches, and luminol is released to produce chemiluminescence in the wide linear range [110].

In another approach, a label-free biosensor was developed for detecting the *I27L* gene variant that is responsible for diabetes. Luminol was used as a chemiluminescence illuminant for the output signal. AuNPs were decorated with the substrate indium tin oxide. The ITO electrode biosensor demonstrated high sensitivity toward mismatched DNA. The electrode was functionalized with the polymer along with AuNPs using oligonucleotides as a capture probe. As soon as the target DNA was hybridized, there was a significant increase in the anionic charge of the electrode that further caused quenching of ECL intensity [111].

A novel ECL-based concept was used for the detection of SNP. A luminol-H_2_O_2_-horseradish peroxide (HRP) system mimicking DNAzyme-fluorescein chemiluminescence resonance energy transfer (CRET) magnetic NPs was developed. The quantitative analysis of SNPs was possible by positive mutation detection. Furthermore, the imaging strategy amplified the signal upon SNP detection along with chemiluminescence because of luminol (https://doi.org/10.1016/j.bios.2014.10.025) (accessed on 20 May 2021).

This novel luminol-based biosensor has also been investigated for other biological functions such as cancer cell detection. Luminol in Ag-PAMAM biocomposite was one such approach based on ECL [112].

Molecular imaging technology has also been of interest among the chemiluminescence-based approaches. Hydrogel polymers with molecular beacon (mb) probes were utilized to form the microgels. By tuning the spatial distribution of molecular probes immobilized on the microgel, it was possible to detect miRNA (miR-21) targets in human serum. The system was highly sensitive as it was able to quantify even the lowest mutant target in a sample of 20 µL. This approach can be further developed for biosensing with other probes, such as aptamers [113].

Based on the aggregation-induced emission (AIE), a molecule TPBT, was used to identify the dsDNA. A dual-color fluorescent signal in the red (~640 nm) and green zone (~537 nm) was emitted. When the molecule binds with dsDNA and ssDNA, there was an emission of red color. On the contrary, green light was emitted only in response to the binding of TPBT with dsDNA exclusively. Hence, this made it possible to detect SNPs in the damaged or mutated DNA with ultrahigh sensitivity. The method was a completely label-free, robust, AIGen-based dsDNA assay [114].

The molecular fluorescence method has been in constant exploration for miRNA detection via using a DNA probe (MB1). A hybridization chain reaction (HCR) was used to develop DNA-based Ag nanoclusters. MB1 contains a poly-cytosine nucleotide loop. An HCR monomer (MB2) with a poly-guanine nucleotide sticky end was developed. Both the monomers co-existed in the solution until the SNP was detected, leading to decreased fluorescence signal. This label-free method allowed the detection of polymorphism in let-7a (miRNA) [115].

This ECL technology is up-and-coming in discovering SNPs and can be further characterized in terms of safety, application protocol, and detection duration. With more improvement, the technique can offer unlimited applications in the field of diagnosis in human health. A few other diagnostic platforms designed on the concept of nanodimensions are mentioned in Table 1.

### 4.3. Nanosheets

**Figure 3:**
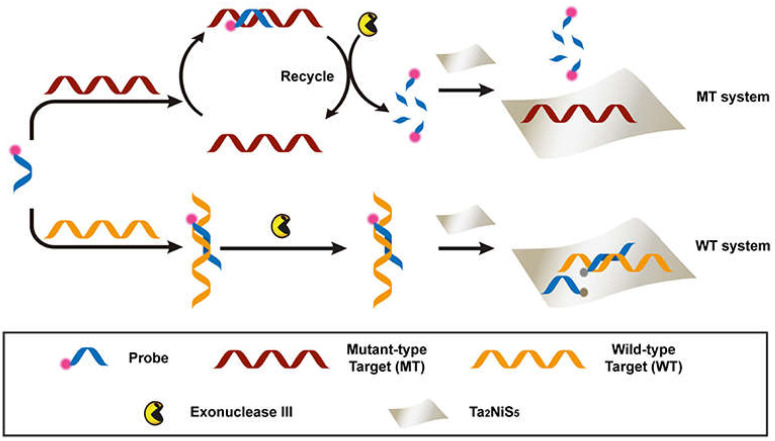
A scheme of the fluorescent sensor composed of a Ta_2_NiS_5_ nanosheet to detect SNPs. Reprinted from ref. [128], Copyright © 2019 under the terms of the Creative Commons Attribution License (CC BY) (accessed on 24 April 2021).

Nanomaterials have been extensively explored in the detection and diagnosis of pathologies in humans [124,125,126]. One such approach based on nanomaterials uses single or multilayered nanosheets made of transition metal dichalcogenide (TMD). TMD nanosheets have various features, including electronic, chemical, and optical properties [127]. A recent study has introduced Ta_2_NiS_5_ nanosheets as an amplification platform for SNP detection [128]. Dye-labeled DNA has been used as a probe to detect the mutant-type target. The ternary chalcogenide nanosheet presents selectivity for the specific oligonucleotides of various lengths by quenching the fluorescence of dye-labeled DNA probes. This methodology is highly sensitive to a single-base mismatch among the wild-type DNAs. Ta_2_NiS_5_ was used as the target amplification biosensing tool. A wild-type (WT) target was used to distinguish the SNP whereas, a dye-labeled DNA was used as a probe (P) for the identification of mutant type targets (MTs), as shown in Figure 3.

In another similar concept, a two-dimensional nanomaterial made of black-phosphorous nanosheets (BPs) was developed for biosensing. The surface-to-volume ratio of BPs can be enhanced by conjugation with functionalization molecules to improve the detection ability. Hence, nitrophenyl was used to enhance the selectivity power of BPs against ctDNA. The fluorescent-labeled ssDNA probe could be attached to the surface of nitrophenyl-functionalized BPs in the absence of the target ctDNA. The nitrophenyl BPs had higher quenching power toward ssDNA compared with double-stranded DNA. A highly efficient method increased the fluorescence up to 5.4 fold when the dye-labeled ssDNA probe formed the dsDNA in the presence of the target ctDNA. This particular platform can be used as a tool for diagnosing SNPs in the blood serum of cancer patients [129].

In the latest diagnostic studies regarding SNPs, graphene oxide (GO) has gained popularity. GO has been utilized in the amplification of the SNP detection signals. Normally, short dye-labeled DNA probes are used in GO applications as the short probes have low fluorescence ratios between complementary DNA and the sequence of SNP. The fluorescence of an unhybridized probe in the presence of a single-base mismatch can be quenched by GO. On the contrary, the fluorescence was observed in the presence of hybridization of dsDNA with complementary target DNA. However, ideally, this is an impractical approach as the target sequences are usually longer, and GO has low fluorescence quenching ability for long-stranded ssDNA compared with short ssDNA. To understand the methodology of GO-based fluorescent nanosheets, a moderate sequence of DNA was used as a probe, whereas the complementary target was a portion of the *Escherichia coli* gene sequence. The fluorometric-DNA-modified GO nanosheets exhibited excellent biosensing properties against the single-base mismatch. The targets had higher fluorescence when the dye was tagged at the 5′ end of the probe, and the mismatched base was near the 5′ end than when it was near the 3′ end. Altogether, the results were highly sensitive to the single-base mismatch, and the results were similar for both the short and long target DNAs [130].

In another study, metallic and semiconductor MoS_2_ nanosheets were fabricated for the fluorescence quenching against the fluorophore-labeled ssDNAs. MoS_2_ nanosheets were exploited to detect SNPs based on the principle of fluorescence quenching and chemical exfoliation. The ultrathin nanosheets made of metallic MoS_2_ had higher quenching efficiency compared with the semiconductor MoS_2_ nanosheets. The multiplex detection biosensor was used to discriminate the ssDNA and dsDNA labeled with various fluorophores efficiently in one solution with high precision [131].

A similar study explored the potential of four TMD nanosheets of WS_2_, MoS_2_, WSe_2_, and MoSe_2_ based on a metal-ion-induction approach. Their specificity was assessed against the ssDNA and dsDNA, and the growth of the nanosheets improved along with thickness with an increase in the amount of metal ion concentration. However, the conjugation between ssDNA and TMD nanosheets was disturbed when the ssDNA hybridized with complementary DNA. The nanosheets were found to be dispersed with a higher metal ion concentration. This feature then enhanced the detection of SNPs with accuracy by the label-free calorimetric approach [132]. Therefore, based on all these studies, it can be stated that nanomaterials designed as nanosheets hold potential in detecting SNPs and need further exploration for toxicity evaluation and clinical translation.

### 4.4. Miscellaneous Nanobased Detection of SNPs

**Figure 4:**
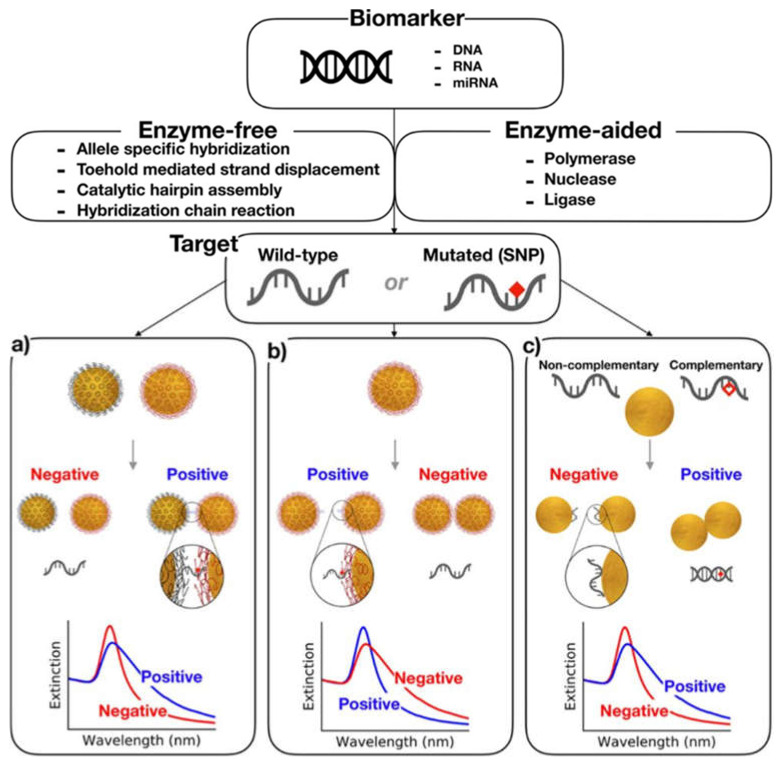
AuNP-based colorimetric assays. (**a**) Crosslinking hybridization assay leading to color change upon the detection of specific DNA hybridization. (**b**) Non-crosslinking method causing the aggregation of the NPs in the absence of a complementary target. (**c**) Unmodified NP-based colorimetric assay in which the ssDNA stabilizes the AuNPs against aggregation, and NPs undergo aggregation in the presence of dsDNA. Reprinted from ref. [136], under the Creative Commons Attribution License (https://creativecommons.org/licenses/by/4.0) (accessed on 24 April 2021).

Various other nanobased systems have been developed, characterized, and explored [133,134,135]. Because of the colloidal stability and electro-optical properties, AuNPs have gained attention in the past for diagnostic purposes. The conductive electrons in the metallic core undergo oscillations in the presence of an external electromagnetic field that generates surface plasmon resonance. This makes the AuNPs useful in biosensing with a high absorption index and almost no photobleaching. Figure 4 summarizes the idea of the detection of DNA damages in different approaches. Figure 4a shows the concept of aggregation upon subsequent hybridization of ssDNA adhered on AuNPs with target oligonucleotide. The method is accurate and reports no false positives. Figure 4b shows the nonselective aggregation of one type of DNA-coated AuNP based on the non-crosslinking method to detect SNP. The presence of a single point mutation at the 5′ end provides colloidal stability to the NPs due to the steric effect. However, the gradual aggregation occurs in the presence of a perfect-match sequence by decreasing steric repulsion. Figure 4c shows the use of citrate-stabilized AuNPs instead of DNA-coated NPs. The citrate-stabilized AuNPs have a high affinity for the ssDNA compared with dsDNA. This might be because ssDNA has high electrostatic linkage with AuNPs due to elevating the number of functional groups [136].

AuNPs stabilized with DNA have also been studied extensively for use in SNP detection. The single-base mismatch can easily be detected among the wild-type DNA fragments. AuNPs have expressed excellent results in liquid biopsies. DNA-coated AuNPs can discriminate the SNP in less than 10 min without the need for amplification. The NPs were incubated with the liquid sample, and the solution remained un-aggregated in the presence of the target sequence [137].

An ultrasensitive DNA biosensor was developed that used DNA-templated Ag deposition from AgNPs along with electrochemical atom transfer radical polymerization signal amplification (eATRP). The target DNA was successfully captured when the thiol-functionalized peptide nucleic acid (PNA) probe was used, modified as an Au electrode. The DNA template was bound with a PNA probe based on the phosphate group functionalization of the thiol via an initiator (α-bromophenylacetic acid). The AgNPs deposited on the electrode, and the concentration of AgNPs proportional to the DNA template were quantified using differential pulse voltammetry. This modification facilitated the detection of SNPs with high sensitivity [138].

**Figure 5:**
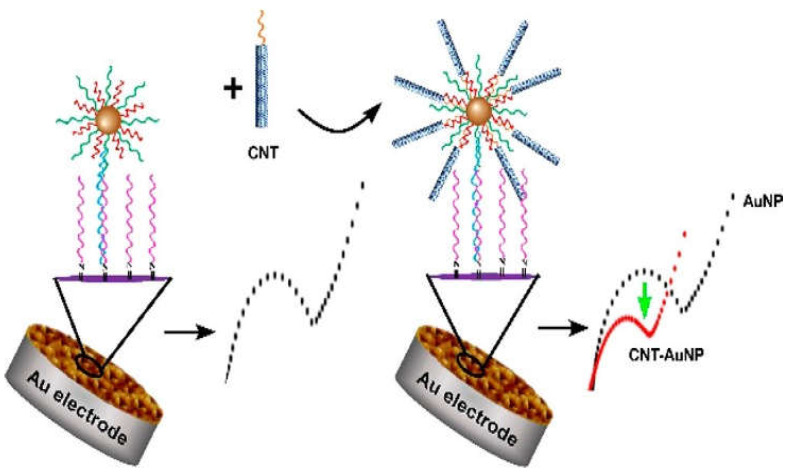
Schematic illustration of carbon nanotubes conjugated with AuNPs, as a Au electrode for DNA probe immobilization. Reprinted with permission from ref. [139]. Copyright 2020 American Chemical Society.

Another novel modality was using an ultrasensitive electrochemical method based on an urchin-like carbon nanotube–AuNP conjugate (CNT-AuNP) nanoclusters. Dopamine was used to modify the Au electrode for DNA probe immobilization. The DNA-functionalized AuNPs were introduced in the sensing system via DNA hybridization after detecting the target. The electrochemical signal was generated when the CNT with DNA was linked to the AuNPs (Figure 5). The 3D nanostructure presented a high sensitivity for the detection of SNPs under optimal conditions [139].

Mesoporous silica NPs have also been explored for the detection of SNPs in the case of β-thalassemia. A detection probe based on mesoporous silica NPs loaded with fluorescein molecules was developed to detect IVS110 point mutation (A > G reversion). Their hybridization with PCR-amplified ssDNA targets yielded different fluorescent signals for mutated targets [140]. The surface plasmon resonance method was another widely explored platform for detecting SNPs. One such use was the identification of the *APOE* gene that is linked with Alzheimer’s disease. The absence of a single-base mismatch to the pre-immobilized biotinylated probes began the cleavage by the *HhaI* restriction enzyme. However, the cleavage was prevented in the presence of the single-base mismatch. The procedure was label-free and straightforward and provided sensitive detection of the SNP linked to Alzheimer’s disease [141].

More unique methods were explored for the detection of SNPs. The latest study focused on the biosensing method using the MutS protein of a bacteria-merged fiber optic particle plasmon resonance (FOPPR) sensing system. The MutS protein adhered to the AuNPs that were deposited on the surface of optical fiber. The mismatched DNA pair was identified by the MutS protein, causing increased absorption of green light. When the MutS protein adhered on the AuNP’s surface interacted with a dsDNA mismatch, the refractive index increased at the AuNP surface resulting in a significant increase in the sensing sensitivity. MutS protein was explored because it initiates DNA repair in *Escherichia coli* after binding with base-pair mismatched DNA and, hence, can be used to detect SNPs. The strategy successfully rapidly identified the mismatched dsDNA with a label-free approach. The FOPPR method facilitated the sensing of dsDNA with its sensor chip containing a sensor fiber [142].

## 5. Challenges in Detecting SNPs via Nanotechnology-Based Methods

Nevertheless, there are considerable obstacles to overcome, and extra effort will be needed to improve the flexibility and detection efficiency of SNP mutation tests. Multiplexed identification is a significant property for high diagnostic accuracy, and some nanotechnological strategies hold great promise in this regard. However, despite considerable progress, many limitations must be overcome before clinical diagnostic assays can be established. Although some experiments have recorded aM and even zM responsiveness, most of these observations are still in the proof-of-concept phase and were performed in clean buffers. Thus, they have not been evaluated in clinically relevant media such as blood, serum, saliva, or urine yet. Moreover, due to the extreme heavy interference from background DNAs, most assessments have registered a very low SNP specificity ratio (10 fold), rendering them potentially inappropriate for detecting low-abundant disease-related SNPs in the background of wild-type gene/genomic DNA.

Furthermore, the lack of multiplexing capacity may be a major challenge to realizing the clinical potential. Another significant consideration for nano-enabled SNP biosensors is the sustainability of biofunctionalized nanomaterials. In this aspect, a thorough understanding of biomolecule–nanomaterial interactions is critical to address issues with high background signals caused by nonspecific adsorptions in serum and/or other complex clinical samples. These also call for enhancing the assays’ automation and miniaturization capabilities, as most existing assays still require using costly and complex equipment and protocols, restricting their capacity for rapid detection.

The current nanotechnology-based diagnostic methods, however, face certain challenges. As nanoparticles can vary from simplest to complex in terms of synthetic methods, their commercialization becomes a problem. Moreover, the reproducibility of each method on a large scale remains questionable. Nanotechnology holds promise in lab settings, but it is difficult to scale up clinically due to certain other factors such as characterization and quality control. The characterization techniques are critical to the evaluation of the diagnostic nanocarriers. Furthermore, the methods should also be validated for the early detection of toxicity of the nanocarriers. Though physical and chemical stability have been studied throughout the phases of various stages of fabrication of NPs, the in vivo biodistribution, absorption, and elimination are still ambiguous. Adequate knowledge of the interaction of the diagnostic nanocarriers with biological membranes is also a question that needs extensive research prior to clinical translation. Altogether, there are currently no regulatory guidelines available for the commercialization of nanotechnology-based diagnostic approaches. Furthermore, the clinical study design needs a thorough evaluation to recruit the nanoproducts to study the toxicity/safety to the population.

In addition, existing nano-biosensing technology-based SNP detection methods tend to work well only under some DNA sequence conditions with short DNA and can only recognize a specific sequence and, therefore, are not suitable for universal SNP detection. Several ingenious ways to design nanomaterials for signal amplification strategies have also been developed, including the use of multiple tagging and enrichment of nanoparticle probes with signal moieties to enable ultrasensitivity. Some of these strategies also hold great promises for multiplexed detection, an essential property for high diagnostic accuracy.

## 6. Conclusions and Outlook

For many decades, PCR methods have been used for detecting SNPs or point mutations. Over the past few years, however, remarkable advances have been made in designing biodiagnostic tools for detecting low-abundance SNPs/mutations and genetic mutations with higher sensitivity and accuracy, mainly in terms of incorporating more advanced DNA amplification methods with biofunctionalized nanomaterials. The significance of these advances is that these new strategies, including NP-target-assisted PCR (or probe) amplification, NP-enhanced signal amplification, target cycling coupled with probe amplification, etc., allow detection of low-concentration SNPs down to the aM–zM range and suggest efficient approaches to improve screening ability from limited DNA material via multiplex PCR methods. Based on the extensive research, it can be concluded that NPs facilitate the implementation of assays to detect genetic defects from the biological samples. Coupling the NPs with DNA-based amplification tools can further improve the sensitivity and precision of the detection. This can be helpful in real-time assays, and with further studies, it can be incorporated into the point-of-care diagnosis. Miniaturization and automation are possible with electrochemical signal transduction, but these approaches are vulnerable to false positives. Recent developments in microfluidics may be able to help SNP assays overcome sample throughput and automation issues. If we can overcome these obstacles, automation of these ultrasensitive assays may lead to integrating sample processing, quantitative determination, and signal measurement in a single system in a real-world clinical environment. This would make quick and precise disease diagnosis and prognosis much easier. As a result, these are still important issues that must be resolved, and further work will be needed to enhance the analytical sensing efficiency and portability of SNP experiments.

## Figures and Tables

**Table 1:** Nanotechnology-based methods for SNP detection.

Technique	Gene/Sequence Detected	Disease	Sensitivity	Outcomes	Ref.
Nanobased ligation assay	*IVSII-1* (G > A)	β-thalassemia	Frequency of 72% for IVSII-1 (G > A) mutation (42% heterozygote, and 30% mutant homozygote) was detected	Excellent sensitivity for allele frequency of IVSII-1 (G > A) mutation in 50 β-thalassemia patients	[116]
Electroactive graphene oxide nanoplatelets	Mismatch sequence	Alzheimer’s disease	A 26% increase in the electrochemical signal for mutant sequence in 5′-ATGGAGGACGTGCGCGGCCGCCTGGT-3 was observed	Discrimination of SNPs efficiently	[117]
Surface-enhanced Raman spectroscopy using AgNPs	Human mitochondrial DNA(16189T → C)	Pancreatic carcinoma	An extremely low level of detection for mitochondiral DNA polymorphism (16189T → C) was found corresponding to extractions from 200 nL of suspension with 120 pancreatic carcinoma cells	Detection of Ag^+^ ions from AgNPs with ion-mediated cascade amplification	[118]
Gallium plasmonic NPs on silica substrate	A single 12-mer sequence from the *H. pylori* (HP1-SH) and 100-mer sequence from exon 11 of the cystic fibrosis transmembrane conductance regulator gene	Cystic fibrosis	Detection of F508del, a three-nucleotide (CTT) deletion at the 508 position, in large genomic DNA isolated from blood cells and *H. pylori* SNP detection among other pathogens from the concentration as low as a few nanomoles with reduction in energy shift	DNA sensing was demonstrated by immobilizing the thiolated capture probe sequence from the *Helicobacter pylori* sequence and single gene mutation in cystic fibrosis onto the substrate	[119]
Kelvin probe force microscopy of DNA-capped NPs	*BRCA1* gene	-	Label-free detection of single-point mismatched DNA (5′-CAGAAAATA AAGGTAG-3′) from *BRCA1* gene	Precise detection of SNPs	[120]
Surface-enhanced Raman spectroscopy (plasmonics nanoprobes)	*BRCA1* gene	Breast cancer	Detection of single-base variation (A/G) at site N47 on the BRCA1 gene that leads to an SNP at codon 504	Specific and selective detection of SNPs by using short DNA probes	[121]
Single microsphere binding AuNPs	*HIV-2* DNA and *KRAS* gene	-	Detection of mutation at one nucleotide in sequence, TTGCCTACGCCATCAGCTCCAACT with precision as compared to wild DNA sequence, TTGCCTACGCCACCAGCTCCAACT	High selectivity to identify mutant DNA from wild-type DNA differing by one nucleotide in 21 nucleotide sequence	[122]
Graphene oxide and AuNPs dual-platform (Surface-enhanced Raman spectroscopy)	A target sequence in DNA	Universal applications, including cancer	The lowest limit of detection as low as 10 fM was achieved for single-nucleotide base mismatch in the DNA (5′TGAAGGATTAGGCAAGTGCCTAGTAATGATC3) discriminating it from the closely related six nontarget DNA sequences	High sensitivity for single-nucleotide base mismatch	[123]

## Data Availability

Data are included within this article.
